# Evaluation of Open Rives-Stoppa and Lichtenstein Repair Methods for Bilateral Inguinal Hernias: A Single-Centre Comparative Analysis

**DOI:** 10.7759/cureus.67946

**Published:** 2024-08-27

**Authors:** Lajpat Rai, Syed Daniyal Raza, Chon Sum Ong, Ali Naqi, Nazish Iftikhar, Ghina Awais, Rutaba Alam, Sheeraz S Siddiqui, Ghina Shamsi, Nazia Lodhi

**Affiliations:** 1 Department of General Surgery, Indus Hospital and Health Network, Karachi, PAK; 2 Department of General Surgery, Ysbyty Gwynedd Hospital, Bangor, GBR; 3 Department of General Surgery, Dow University of Health Sciences, Dow International Medical College, Karachi, PAK

**Keywords:** bilateral inguinal hernia, early complications, hernia recurrence, lichtenstein, myopectineal orifice, pre-peritoneal approach, rives-stoppa, types of hernia repair

## Abstract

Aim

The purpose of the study is to compare the two common open surgical methods for bilateral inguinal hernias: bilateral Lichtenstein repair and Rives-Stoppa repair. It evaluates their benefits, drawbacks, and outcomes to improve the management of bilateral inguinal hernias and enhance patient care and results.

Background

Abdominal wall hernias are prevalent in the surgical field, and they occur when intra-abdominal organs protrude through weakened or torn regions in the abdominal wall. The Lichtenstein repair, also known as the tension-free mesh repair, is one of the most widely used techniques that involves placing a synthetic mesh over the hernia defect to reinforce the abdominal wall. The Rives-Stoppa technique takes the posterior approach, which involves placing a large mesh in the preperitoneal space, which provides broad coverage of the potential hernia sites.

Method

This retrospective study included 86 male patients from the Department of General Surgery at Indus Health Network, Karachi, Pakistan. Data were collected up to three months post-operation for all open bilateral inguinal hernia repairs performed between January 2017 and April 2021. The patients were divided into two groups: group A underwent Lichtenstein repair, while group B underwent Rives-Stoppa repair. The procedures were performed by different surgeons and surgical trainees under direct supervision.

Results

Regarding post-operative complications (scrotal swelling, epididymo-orchitis, seroma formation, ipsilateral testicular swelling, surgical site infection, erectile dysfunction, wound dehiscence, fever, hydrocele, sensory abnormality, hernia recurrence in 3 months, post-operative pain in 14 days), there was no significant difference observed between the two groups. There were two recurrences within three months after Lichtenstein repair and one recurrence after Stoppa repair, but no statistical difference was demonstrated.

Conclusion

Statistically, both the Lichtenstein repair and the Rives-Stoppa repair demonstrated similar outcomes. However, the Rives-Stoppa repair offers distinct advantages for bilateral inguinal hernia repair, making it a preferable option in many cases as this approach utilises a single midline incision, simultaneously facilitating access to both hernial sites. This method ensures complete coverage of the myopectineal orifices bilaterally, addressing all potential hernia sites in the lower abdomen. These features collectively contribute to the technique's efficacy in managing bilateral hernias.

## Introduction

Abdominal wall hernias are commonly encountered in the surgical speciality, with a prevalence of 1.7% for all ages and 4% for those aged over 45 years. Inguinal hernias occur when intra-abdominal organs protrude through weakened or torn regions in the abdominal wall. They account for 75% of abdominal wall hernias, with a lifetime risk of 27% in men and 3% in women [1]. If left untreated, inguinal hernias can lead to life-threatening complications like bowel obstruction or strangulation, which require emergency surgical intervention. The risk factors for developing an inguinal hernia vary. These include patient risk factors, such as male gender, old age, a patent processus vaginalis, and external factors like increased intra-abdominal pressure [2].

Therefore, the choice of surgical technique for hernia repair is a critical decision that can significantly impact patient outcomes, which encompass post-operative pain levels, recurrence rate, recovery duration, and more [3]. The optimal technique should be tailored to the patient’s characteristics, hernia type, surgeon expertise, and preferences to maximise efficacy and minimise complications. The surgeons have multiple options, including open repair techniques like the Rives-Stoppa and Lichtenstein techniques and minimally invasive repair techniques such as laparoscopic or robotic hernia repair. The decision between open and minimally invasive repair techniques is influenced by factors like the patient’s overall health, the surgeon’s expertise, and specific hernia characteristics.

The Rives-Stoppa technique, also known as the Stoppa procedure, involves a pre-peritoneal approach with the placement of a large prosthetic mesh to reinforce the entire myopectineal orifice bilaterally. The myopectineal orifice, first described by the French surgeon Fruchard, includes the medial, lateral, and femoral triangles, which are potential sites for groin hernias. The students of Fruchard, Stoppa, and Rives subsequently developed the pre-peritoneal approach to hernia repairs [4]. In contrast, the Lichtenstein repair utilises an anterior approach, applying smaller meshes to reinforce each inguinal canal individually. The tension-free repair technique reinforces the weakened posterior wall with mesh without directly repairing it. The standardisation by Lichtenstein remains the gold standard for managing unilateral inguinal hernia repair [5].

Evidence-based selection of repair techniques is fundamental to achieving optimal short-term and long-term results in hernia surgery. The primary objective of this study is to perform a comprehensive comparative analysis of two commonly used open-surgical methods for treating bilateral inguinal hernias: bilateral Lichtenstein repair and Rives-Stoppa repair. By carefully examining the benefits, drawbacks, and outcomes, this study aims to provide insights into managing bilateral inguinal hernias, particularly post-operative pain and hernia recurrence rates. Using this information, surgeons can make informed decisions and effectively communicate options to patients when selecting between the Lichtenstein and Rives-Stoppa repairs, ultimately enhancing patient care and improving outcomes.

## Materials and methods

This retrospective study involved 86 male patients who attended the Department of General Surgery, Indus and Health Network, Karachi, Pakistan. The data were collected up to three months after the operation for all open bilateral inguinal hernia repairs performed between January 2017 and April 2021. The patients were divided into two groups: group A, which had Lichtenstein repair, and group B, which had Rives-Stoppa repair done. The procedures were performed by different surgeons and surgical trainees of various training levels under direct supervision. All the procedures were performed under a mixture of general and spinal anaesthesia, and all patients received prophylactic antibiotics.

The inclusion criteria of this study were male patients aged between 14 and 80 years old with bilateral primary or recurrent direct or indirect inguinal hernia, an open approach, and elective repairs. The exclusion criteria were patients of other ages not within the stated range, female gender, unilateral inguinal hernias, laparoscopic approach, and emergency repairs.

The pre-operative body mass index (BMI), occupation, co-morbidities, smoking status, and previous history of inguinal hernia repair were recorded. The predisposing factors to hernia, including the history of weight lifting, transurethral resection of the prostate (TURP), chronic cough, lower urinary tract symptoms, and constipation, were noted.

Lichtenstein tension-free hernioplasty

The surgical approach began with an inguinal skin incision positioned 2 cm superior and parallel to the inguinal ligament. This incision extended from a point superior and lateral to the pubic tubercle to a point inferior and medial to the anterior superior iliac spine. For indirect hernias, the sac was carefully dissected and then ligated using a Vicryl 2/0 suture before being sectioned. In cases of large direct hernias, the sacs were invaginated and plicated using a Prolene 2/0 suture. A standard-sized Prolene mesh measuring 6 cm × 11 cm was utilised in all procedures. The mesh was secured in position using polypropylene 2/0 sutures. The fixation process involved attaching the mesh inferiorly to the inguinal ligament and superiorly to the conjoint tendon, ensuring coverage from the pubic tubercle to a point beyond the internal ring orifice. The wound was then closed in layers [6].

Rives-Stoppa procedure

A lower midline incision was used to access the abdominal space. The subcutaneous fat and rectus sheath were incised, and the rectus muscles were separated along the midline. The pre-peritoneal space was carefully dissected, extending from the retropubic space of Retzius to the rectus abdominis muscles and further into the retro-inguinal space. The spermatic cord and gonadal vessels were identified during this process. Key anatomical landmarks, including the superior pubic rami, obturator foramens, and iliac vessels, were visualised. Small direct and indirect hernia sacs were dissected and reduced. For large indirect hernias, the sacs were divided; the proximal portion was sutured with Vicryl 3/0, and the distal part was left in situ. Parietalisation of the spermatic cord and gonadal vessels was performed by dissection of their peritoneal attachments. A large polypropylene mesh (30 cm × 30 cm) was reshaped and positioned over the peritoneum. The superior margin was just below the umbilicus, inferiorly the retropubic space, and laterally, the external iliac vessels. The procedure concluded with the layered closure of the wound [7].

Follow-up

The patients were routinely followed up two weeks after the surgery in the clinic and more after that if deemed required. The patients were asked a series of questions, and the data were stored in the local hospital's electronic record.

Statistical analysis

Statistical analysis was performed using the Statistical Package for the Social Sciences (SPSS, IBM Corp., Armonk, NY) software. The comparison between different groups in terms of categorical variables was performed using the chi-square test. The significance level was set at a P-value of <0.05.

Ethics approval

This study was conducted in accordance with the ethical standards of the Indus Hospital and Health Network (IHHN) Institutional Review Board (IRB). The ethics approval for this research was obtained from the Indus Hospital and Health Network Institutional Review Board. The IRB number is IHHN_IRB_2021_04_017.

## Results

The study had 86 male patients in total. Group A included 35 (41%) patients who underwent elective Lichtenstein repair, whereas group B included 51 (59%) patients who underwent the Stoppa procedure. For group A, the median age of the patients is 63 years, the youngest being 48 years, and the oldest being 68 years; for group B, the median age is 60 years, the youngest being 50 years, and the oldest being 67 years. Regarding pre-operative data, no statistically significant differences were found between the two groups except for occupation (Table 1).

**Table 1:** Pre-operative data of the patients undergoing Lichtenstein or Stoppa’s repair

Variables	Lichtenstein repair = N (%)	Stoppa repair = N (%)	Total = N (%)	P-value
Pre-operative BMI				0.705
Under-weight	7 (20.0)	6 (11.8)	13 (15.1)	-
Normal	10 (28.6)	19 (37.3)	29 (33.7)	-
Overweight	6 (17.1)	9 (17.6)	15 (17.4)	-
Obese	12 (34.3)	17 (33.3)	29 (33.7)	-
Occupation				0.013
Professional	1 (3.6)	1 (4.8)	2 (4.1)	-
Retired	5 (17.9)	0 (0)	5 (10.2)	-
Skilled worker	13 (46.4)	9 (42.9)	22 (44.9)	-
Unskilled worker	5 (17.9)	11 (52.4)	16 (32.7)	-
Unemployed	4 (14.3)	0 (0)	4 (8.2)	-
Presence of comorbidities				0.47
Yes	12 (34.3)	13 (25.5)	25 (29.1)	-
No	23 (65.7)	38 (74.5)	61 (70.9)	-
1. Hypertension				0.796
Yes	9 (25.7)	11 (21.6)	20 (23.3)	-
No	26 (74.3)	40 (78.4)	66 (76.7)	-
2. Diabetes mellitus				0.696
Yes	2 (5.7)	5 (9.8)	7 (8.1)	-
No	33 (94.3)	46 (90.2)	79 (91.9)	-
3. Congestive heart disease				0.644
Yes	1 (2.9)	4 (7.8)	5 (5.8)	-
No	34 (97.1)	47 (92.2)	81 (94.2)	-
4. Psychiatric illness				0.407
Yes	1 (2.9)	0 (0)	1 (1.2)	-
No	34 (97.1)	51 (100.0)	85 (98.8)	-
5. Asthma				0.407
Yes	1 (2.9)	0 (0)	1 (1.2)	-
No	34 (97.1)	51 (100.0)	85 (98.8)	-
6. Hepatitis C				0.407
Yes	1 (2.9)	0 (0)	1 (1.2)	-
No	34 (97.1)	51 (100.0)	85 (98.8)	-
Smoking status				0.362
Ex-smoker	3 (8.6)	6 (11.8)	9 (10.5)	-
Current smoker	9 (25.7)	7 (13.7)	16 (18.6)	-
Non-smoker	23 (65.7)	38 (74.5)	61 (70.9)	-
Previous history of inguinal hernia repair				0.559
None	29 (82.9)	41 (80.4)	70 (81.4)	-
Bilateral inguinal hernia	1 (2.9)	2 (3.9)	3 (3.5)	-
Unilateral left-sided Inguinal hernia	1 (2.9)	5 (9.8)	6 (7.0)	-
Unilateral right-sided Inguinal hernia	4 (11.4)	3 (5.9)	7 (8.1)	-

Table 2 depicts the risk factors of the patients who underwent Lichtenstein and Stoppa repairs. The common risk factors that predispose patients to hernia were included (history of weight lifting, TURP, chronic cough, lower urinary tract symptoms, and constipation). The history of lower urinary tract symptoms was also broken down into poor urinary systems, intermittency, dribbling, a sense of incomplete urination, hesitancy, and retention. Comparing the risk factors, statistically significant differences were only shown for the history of chronic cough in the Lichtenstein group and the history of intermittency in the Stoppa group. For most other variables, there was no significant difference between the Lichtenstein and Stoppa repair groups, suggesting that the two groups were relatively similar concerning these risk factors and symptoms.

**Table 2:** Risk factors predisposing to hernia development of the patients undergoing Lichtenstein or Stoppa repair

Risk factors	Lichtenstein repair = N (%)	Stoppa repair = N (%)	Total = N (%)	P-value
History of weight-lifting				0.176
Yes	16 (45.7)	16 (31.4)	32 (37.2)	-
No	19 (54.3)	35 (68.6)	54 (62.8)	-
History of TURP				0.137
Present	6 (17.1)	16 (31.4)	22 (25.6)	-
Absent	29 (82.9)	35 (68.6)	64 (74.4)	-
History of chronic cough				0.000
Present	9 (25.7)	0 (0)	9 (10.5)	-
Absent	26 (74.3)	51 (100.0)	77 (89.5)	-
History of lower urinary tract symptoms (LUTS)				
(a) Poor urinary stream				0.215
Present	7 (20.0)	5 (9.8)	12 (14.0)	-
Absent	28 (80.0)	46 (90.2)	74 (86.0)	-
(b) Intermittency				0.038
Present	29 (82.9)	49 (96.1)	78 (90.7)	-
Absent	6 (17.1)	2 (3.9)	8 (9.3)	-
(c) Dribbling				0.115
Present	5 (14.3)	2 (3.9)	7 (8.1)	-
Absent	30 (85.7)	49 (96.1)	79 (91.9)	-
(d) Sense of incomplete urination				0.393
Present	3 (8.6)	2 (3.9)	5 (5.8)	-
Absent	32 (91.4)	49 (96.1)	81 (94.2)	-
(e) Hesitancy				0.083
Present	7 (20.0)	3 (5.9)	10 (11.6)	-
Absent	28 (80.0)	48 (94.1)	76 (88.4)	-
(f) Retention				0.163
Present	2 (5.7)	0 (0)	2 (2.3)	-
Absent	33 (94.3)	51 (100)	84 (97.7)	-
History of constipation				0.071
Present	22 (62.9)	41 (80.4)	63 (73.3)	-
Absent	13 (37.1)	10 (19.6)	23 (26.7)	-

As to the early post-operative complications (scrotal swelling, epididymal-orchitis, seroma formation, ipsilateral testicular swelling, surgical site infection, erectile dysfunction, wound dehiscence, fever, hydrocele, sensory abnormality, hernia recurrence in three months, post-operative pain in 14 days), there were no statistically significant differences. Despite this, five (14.3%) patients developed seroma following a Lichtenstein repair, while only three (5.9%) patients did after a Stoppa procedure, indicating an 8.4% difference. The data also suggested that the Stoppa procedure was associated with a lower incidence of surgical site infection in only two (3.9%) patients compared to the Lichtenstein repair group, which had five (14.3%) patients. There were two recurrences within three months after the Lichtenstein repair and one recurrence after the Stoppa repair, but no statistical difference was demonstrated (Table 3).

**Table 3:** Post-operative complications of the patients after Lichtenstein or Stoppa repair

Variables	Lichtenstein repair = N (%)	Stoppa repair = N (%)	Total = N (%)	P-value
1. Scrotal swelling				0.3
Present	32 (91.4)	50 (98.0)	82 (95.3)	-
Absent	3 (8.6)	1 (2.0)	4 (4.7)	-
2. Epididymo-orchitis				0.407
Present	1 (2.9)	0 (0)	1 (1.2)	-
Absent	34 (97.1)	51 (100.0)	85 (98.8)	-
3. Seroma formation				0.262
Present	5 (14.3)	3 (5.9)	8 (9.3)	-
Absent	30 (85.7)	48 (94.1)	78 (90.7)	-
4. Ipsilateral testicular swelling				0.407
Present	1 (2.9)	0 (0)	1 (1.2)	-
Absent	34 (97.1)	51 (100.0)	85 (98.8)	-
5. Surgical site infection (SSI)				0.115
Present	5 (14.3)	2 (3.9)	7 (8.1)	-
Absent	30 (85.7)	49 (96.1)	79 (91.9)	-
6. Erectile dysfunction				0.407
Present	1 (2.9)	0 (0)	1 (1.2)	-
Absent	34 (97.1)	51 (100.0)	85 (98.8)	-
7. Wound dehiscence				1
Present	0 (0)	1 (2.0)	1 (1.2)	-
Absent	35 (100.0)	50 (98.0)	85 (98.8)	-
8. Fever				0.407
Present	1 (2.9)	0 (0)	1 (1.2)	-
Absent	34 (97.1)	51 (100.0)	85 (98.8)	-
9. Hydrocele				1
Present	1 (2.9)	2 (3.9)	3 (3.5)	-
Absent	34 (97.1)	49 (96.1)	83 (96.5)	-
10. Sensory abnormalities				0.407
Present, along with the incision	1 (2.9)	0 (0)	1 (1.2)	-
Absent	34 (97.1)	51 (100.0)	85 (98.8)	-
11. Hernia recurrence (3 months)				0.564
Present	2 (5.7)	1 (2.0)	3 (3.5)	-
Absent	33 (94.3)	50 (98.0)	83 (96.5)	-
12. Post-operative pain (at 14 days)				-
Present	0 (0)	0 (0)	0 (0)	-
Absent	35 (100.0)	51 (100.0)	86 (100.0)	-

## Discussion

The factors to be considered when choosing a hernia repair method include recurrence risk, safety, post-operative recovery and quality of life, level of difficulty and reproducibility, and cost [8]. The European Hernia Society has published guidelines for the treatment of inguinal hernia in adults and recommended Lichtenstein repair as the open method of choice for primary bilateral hernia. Stoppa’s repair is the treatment of choice for complex (bilateral or several recurrences) hernias. Nyhus emphasises that modern hernia surgery should be tailored to each clinical situation, with some suggesting it should also consider individual social and economic circumstances [9].

In the present study, the median age of the patients was younger in group A (63 years) and 60 years for group B, which is comparable with the other studies [10,11]. There was no statistically significant difference between bilateral Lichtenstein and Stoppa’s repair regarding post-operative complications. The results are compatible with those of Fernandez et al. [7], Malazgirt et al. [11], and Al-Shemy et al. [4]. The meta-analysis of 10 randomised controlled trials and two comparative studies published by Li et al. has concluded the same: there was no statistical difference in post-operative complications between the Stoppa’s and Lichtenstein techniques [12]. The post-operative pain and hernia recurrence rate were the two parameters that this study focused on.

Post-operative pain

Some authors considered chronic pain to be a more severe complication than hernia recurrence due to its debilitating nature [13]. Chronic pain greatly impacts daily activities, relationships, and overall life enjoyment. Some researchers have proposed that the intensity of early post-operative pain can reliably predict the likelihood of developing chronic pain. The pain scores during the first 14 days showed a strong correlation with pain scores at long-term follow-up [14]. None of the patients in this study suffered from pain 14 days post-operatively when asked in the routine follow-up clinic. A few other studies have also demonstrated no statistical difference in post-operative pain after seven days in both procedures [15-18]. However, Sharma et al. concluded that inguinodynia in Lichtenstein's repair is more compared to Stoppa’s repair. They attributed this to the dissection within the inguinal canal and the fixation of the mesh [19]. This is also supported by other studies showing an increased risk of inguinodynia with Lichtenstein repair, which affects the quality of life [4,20]. The possible explanation is that Stoppa‘s repair, being a posterior preperitoneal procedure, involves dissection in a plane with no nerves and minimal cord handling, thus minimising the risk of inguinodynia and testicular atrophy [21].

Hernia recurrence

With different mesh techniques, recurrences are often observed early in the follow-up period due to technical failures. This is one of the reasons that the follow-up is only limited to three months post-operatively [5]. In this study, there were two recurrences in the patients with Lichtenstein repair and only one in the Stoppa’s repair patients. The difference was not statistically significant. Pelissier et al. did suggest that all recurrences occur through the myopectineal orifice, and Stoppa’s repair recurrence rate is low due to the adequate overlap of the mesh on the hernial defects [22]. This is supported by many other studies [23-25].

While both Stoppa’s repair and bilateral Lichtenstein repair for managing bilateral inguinal hernia show comparative post-operative outcomes, there should be a preference for Stoppa’s repair for multiple reasons. The single incision approach could be cosmetically preferable for the patients, and the comprehensive coverage of the myopectineal orifices addresses all potential sites of weakness in the lower abdomen. The procedure enables the examination and repair of not just the inguinal region but also other possible hernia sites, including the midline incisional hernias. The comprehensive inspection ensures that any additional hernias are detected, and there is an option to treat them during the same surgery. Operative time could also be shortened to perform a single incision rather than two separate Lichtenstein repairs. The strategic placement of the mesh also minimises the risk of nerve injury, neuralgia, orchitis, testicular atrophy, and chronic neuropathic pain. However, there are concerns about the technical complexity of Stoppa’s repair, which might require greater surgical skill and experience. The learning curve may be steeper for surgeons trying to master Stoppa’s technique. Therefore, the choice between the two techniques should be based on patient-specific factors, surgeon expertise, and available resources [16,26].

Limitations

The limitations of this study include the unknown training and experience level of the operating surgeons, as well as the relatively small sample size and short follow-up period. They may affect the generalisability and statistical power of the findings. To address these limitations, future studies should be prospective, involve a larger cohort of participants, and extend the follow-up period to assess long-term complications. Additionally, recording parameters such as operating time for both procedures would provide valuable insights. The study's design and the nature of the retrospective study also present a potential risk of bias due to the unequal number of patients in each group and demographic differences. Selection bias might be present, and that would limit the ability to establish causality. By identifying and mitigating these biases, the accuracy and reliability of the findings can be significantly improved, providing a stronger foundation for determining the superior surgical procedure.

## Conclusions

The comparative evaluation of Rives-Stoppa repair and bilateral Lichtenstein repair techniques has offered valuable insights into the management of bilateral inguinal hernias. Each method has unique benefits and drawbacks that impact surgical outcomes. With comparable outcomes, while Lichtenstein repair provides familiarity and simplicity, Rives-Stoppa repair offers a relatively lower risk of nerve injury, using a single incision and mesh and the effective closure of all myopectineal orifices. These advantages contribute to its overall efficacy and safety. Stoppa’s repair ought to be routinely integrated into the healthcare system for treating hernia patients, particularly in complex cases, and should be included in the professional training curriculum for junior surgeons. These findings are crucial for clinical practice, underscoring the need to consider patient-specific factors, surgeon expertise, and hospital resources when choosing the best surgical approach.
